# Methanotrophy under Versatile Conditions in the Water Column of the Ferruginous Meromictic Lake La Cruz (Spain)

**DOI:** 10.3389/fmicb.2016.01762

**Published:** 2016-11-11

**Authors:** Kirsten Oswald, Corinne Jegge, Jana Tischer, Jasmine Berg, Andreas Brand, María R. Miracle, Xavier Soria, Eduardo Vicente, Moritz F. Lehmann, Jakob Zopfi, Carsten J. Schubert

**Affiliations:** ^1^Department of Surface Waters – Research and Management, Swiss Federal Institute of Aquatic Science and TechnologyKastanienbaum, Switzerland; ^2^Department of Environmental Systems Science, Institute of Biogeochemistry and Pollutant Dynamics, ETH Zurich, Swiss Federal Institute of TechnologyZurich, Switzerland; ^3^School of Architecture, Civil and Environmental Engineering, EPFL, Swiss Federal Institute of TechnologyLausanne, Switzerland; ^4^Department of Environmental Sciences, University of BaselBasel, Switzerland; ^5^Department of Biogeochemistry, Max Planck Institute for Marine MicrobiologyBremen, Germany; ^6^Department of Microbiology and Ecology, Cavanilles Institute of Biodiversity and Evolutionary Biology, University of ValenciaBurjassot, Spain

**Keywords:** ferruginous, meromixis, oxycline, anoxic hypolimnion, methane oxidation, aerobic methanotrophs

## Abstract

Lakes represent a considerable natural source of methane to the atmosphere compared to their small global surface area. Methanotrophs in sediments and in the water column largely control methane fluxes from these systems, yet the diversity, electron accepting capacity, and nutrient requirements of these microorganisms have only been partially identified. Here, we investigated the role of electron acceptors alternative to oxygen and sulfate in microbial methane oxidation at the oxycline and in anoxic waters of the ferruginous meromictic Lake La Cruz, Spain. Active methane turnover in a zone extending well below the oxycline was evidenced by stable carbon isotope-based rate measurements. We observed a strong methane oxidation potential throughout the anoxic water column, which did not vary substantially from that at the oxic/anoxic interface. Both in the redox-transition and anoxic zones, only aerobic methane-oxidizing bacteria (MOB) were detected by fluorescence *in situ* hybridization and sequencing techniques, suggesting a close coupling of cryptic photosynthetic oxygen production and aerobic methane turnover. Additions of nitrate, nitrite and to a lesser degree iron and manganese oxides also stimulated bacterial methane consumption. We could not confirm a direct link between the reduction of these compounds and methane oxidation and we cannot exclude the contribution of unknown anaerobic methanotrophs. Nevertheless, our findings from Lake La Cruz support recent laboratory evidence that aerobic methanotrophs may be able to utilize alternative terminal electron acceptors under oxygen limitation.

## Introduction

Among all greenhouse gases, methane (CH_4_) has shown the highest atmospheric concentration increase (factor of 2.5) since industrialization (Forster et al., 2007) with total emissions currently approximating ~600 Tg CH_4_ a^−1^ (Ehhalt et al., 2001). Although this only constitutes a small proportion compared to carbon dioxide (CO_2_) emissions, methane has a global warming potential which is 20 times higher over a 100 year period (Forster et al., 2007). Methane is not only emitted through anthropogenic activities (50–65%; Ciais et al., 2014), but also by a variety of natural sources. Regardless of its source, about 85% of the global methane budget is produced by methanogenic microorganisms (Knittel and Boetius, 2009) in the final step of organic matter degradation. Freshwater lakes occupy only 2–3% of the global terrestrial surface area (Downing et al., 2006), yet they are estimated to contribute between 8 and 72 Tg CH_4_ a^−1^ (1.3–12%) to total CH_4_ emissions (Bastviken et al., 2004, 2011).

In lakes, methane is principally produced in anoxic sediments by methanogenic archaea. In fully mixed lakes, where oxygen is present throughout the water column, and even penetrates into the upper sediment layers, methane is efficiently eliminated through aerobic oxidation (Bastviken et al., 2002). However, in permanently stratified (meromictic) and frequently also in seasonally stratified lakes (mono- or dimictic), an anoxic hypolimnion can be formed below the oxycline, where CH_4_ can potentially accumulate to high concentrations (Schubert et al., 2010; Blees et al., 2015; Lehmann et al., 2015). Here, other oxidative processes could be important, as is the case in marine environments where anaerobic oxidation of methane (AOM) coupled to sulfate (${\text{SO}}_{4}^{2-}$) reduction is the predominant methane sink (Knittel and Boetius, 2009). Canonical AOM is mediated by anaerobic methanotrophic archaea (ANME) directly (Milucka et al., 2012) or together with a deltaproteobacterial partner (Knittel and Boetius, 2009). Furthermore, AOM coupled to nitrate reduction can also be performed by the novel archaeal clade ANME-2d (Haroon et al., 2013). Evidence for AOM proceeding concurrently with iron or manganese reduction also exists for marine settings (Beal et al., 2009; Wankel et al., 2012; Slomp et al., 2013; Riedinger et al., 2014), but the involved microorganisms have not yet been identified.

Conventional sulfate-coupled AOM is an efficient pathway for CH_4_ oxidation in oceans, but although there is some biogeochemical and microbiological indication of AOM in freshwater systems (Eller et al., 2005; Durisch-Kaiser et al., 2011), it has not been shown to play a significant role in anoxic hypolimnia of lakes. This is likely due to relatively low ${\text{SO}}_{4}^{2-}$ concentrations (μM range) in freshwater environments compared to ~28 mM in the oceans. Instead, methane oxidation (MO) mediated by aerobic methane-oxidizing bacteria (MOB) belonging to the alpha- or gamma-subdivision of the Proteobacteria has been considered the principal pathway for methane removal in lakes (e.g., King, 1992; Hanson and Hanson, 1996). The division of alpha-MOB (type II) and gamma-MOB (type I and type X) is based on functional differences with regards to carbon assimilation and the ability to fix nitrogen (Hanson and Hanson, 1996). Genes encoding for soluble-(sMMO) or particulate methane monooxygenase (pMMO), the principle enzyme involved in MO, are expressed by both alpha- and gamma-MOB (Semrau et al., 2010).

Maximum MO rates and MOB abundances usually occur at the oxic/anoxic interface within sediments or the water column, where gradients of both O_2_ and CH_4_ intersect (Rudd et al., 1976). However, MO in the absence of detectable O_2_ coinciding with populations of predominantly aerobic MOB below the oxycline has been reported for several stratified lakes (Schubert et al., 2010; Biderre-Petit et al., 2011; Blees et al., 2014; Oswald et al., 2016). In shallow lakes with light penetration below the oxycline, aerobic MO may be coupled to *in situ* production of oxygen by photosynthesis (Milucka et al., 2015; Oswald et al., 2015; Brand et al., 2016). Additionally, MO (by facultative aerobic MOB) may be coupled to denitrification under oxygen limitation (Kits et al., 2015a,b). *Methylomirabilis oxyfera* (phylum NC 10), for example, has been shown to couple nitrite reduction to NO with methane oxidation, where O_2_ is produced intracellularly by NO dismutation and used to oxidize methane aerobically (Ettwig et al., 2010). This process is likely also relevant in natural anoxic waters, as has been suggested for lake sediments (Deutzmann et al., 2014). Similarly, both iron and manganese oxides are important electron acceptors in terrestrial and aquatic settings, and geochemical evidence suggests that MO in lakes may also be linked to iron reduction (Sivan et al., 2011; Norði et al., 2013), or the cycling of both metals (Crowe et al., 2011).

More and more studies report on the possible involvement of electron acceptors alternative to O_2_ and ${\text{SO}}_{4}^{2-}$ in methane oxidation below the oxycline of stratified lakes, despite the fact that the microbial community seems to be composed predominantly of aerobic methanotrophs in these lacustrine settings. To further elucidate this apparent paradox, we investigated MO in the ferruginous meromictic Lake La Cruz, Central Spain. Biogeochemical studies thus far have focused on iron-cycling processes in Lake La Cruz (Walter et al., 2014), whereas aspects concerning methane oxidation and the present methanotrophic community remained unaddressed. Due to its peculiar stratification regime, its shallow oxycline, low sulfate but high concentrations of iron, Lake La Cruz represents an ideal system to investigate methane oxidation and its potential coupling to the Fe-cycle. In order to study MO pathways and environmental controls, we examined the water column chemistry, including relevant isotopic signatures (e.g., δ^13^C-CH_4_), conducted experiments to quantify methane oxidation rates, tested the contribution of alternative oxidants (nitrate, nitrite, iron, and manganese) to methane oxidation, and characterized the methanotrophic community using molecular techniques (hybridization and sequencing).

## Methods

### Field site

Lake La Cruz is a small (surface area ~0.015 km^2^) lake situated in Eastern Spain near the city of Cuenca at an altitude of about 1000 m a.s.l. It is an almost circular karstic sinkhole which is fed laterally by subaquatic springs about 4–5 m above the lake bottom (Vicente and Miracle, 1988). The lake has an average depth of 20 m which fluctuates seasonally and based on weather conditions (Rodrigo et al., 2001). A salinity gradient maintains permanent stratification (chemocline at 18–19 m), which was established about 300 years ago (Julià et al., 1998). Lake La Cruz exhibits two stratification regimes: in winter the lake is mixed down to 19 m, whereas in summer an oxycline is formed at around 15 m (Rodrigo et al., 2001). The lake is unique in terms of its unusually high concentrations of dissolved iron(II) in the monimolimnion (Rodrigo et al., 2001; Walter et al., 2014).

### *In situ* profiling

A field campaign was carried out the first week of March, 2015. Profiles were measured from a boat at the deepest part in the center of the lake (39°59′20″ N, 01°52′25″ E). A custom-made profiling *in situ* analyzer (PIA) equipped with a multi-parameter probe and various other sensors was deployed to monitor conductivity, turbidity, temperature, depth (pressure), and pH (XRX 620, RBR), photosynthetically active radiation (PAR; LI-193 Spherical Underwater Quantum Sensor, LI-COR) and chlorophyll a (ECO-FL, Wetlands, EX/EM = 470/695), and dissolved O_2_. The two micro-optodes (PSt1 and TOS7, PreSens) attached to the multi-parameter probe allowed for the detection of dissolved oxygen concentrations of 125 and 20 nM, respectively (Kirf et al., 2014).

### Sample collection

Water samples to determine concentrations of other chemical constituents were pumped to the surface with a peristatic pump (Zimmermann AG Elektromaschinen, Horw, Switzerland) with gas tight tubing (PVC Solaflex, Maagtechnic) attached to the PIA and connected to a conical inlet device (Miracle et al., 1992). To ensure that water was pumped from the appropriate depth, the tubing was flushed for 2 min (time required to replace the entire volume of the tubing) before water was filled directly into a syringe (60 ml) from the tube outlet, taking care that air was not introduced. Water was then distributed into vials with the appropriate preservative. Zinc acetate (final concentration ~1.3%) was used to fix total sulfide (H_2_S+HS^−^). Samples for the determination of dissolved (<0.45 μm, cellulose acetate filter) and total metal species were added directly to Suprapur HNO_3_ (65%, Merck) to a final concentration of 0.1 M. Similarly, HCl (0.5 M final concentration) was used to acidify dissolved (<0.45 μm, cellulose acetate filter) and total fractions of Fe(II)/(III) for photometric determination. Aliquots for nitrate (${\text{NO}}_{3}^{-}$), nitrite (${\text{NO}}_{2}^{-}$), ammonium (${\text{NH}}_{4}^{+}$), phosphate (${\text{PO}}_{4}^{3-}$), sulfate (${\text{SO}}_{4}^{2-}$), dissolved inorganic carbon (DIC), and dissolved organic carbon (DOC) were filtered (<0.22 μm, cellulose acetate filter). For methane analysis, serum bottles (120 ml) were filled anoxically by allowing the water to overflow at least two volumes and adding Cu(I)Cl (~0.15% [w/v] final concentration) to stop microbial activity before closing the bottles (without headspace or bubbles) with butyl stoppers (Geo-Microbial Technologies, Inc.) and aluminum crimp seals.

Water for incubation experiments, catalyzed reporter deposition-fluorescence *in situ* hybridization (CARD-FISH) and DNA analysis was retrieved with a Niskin bottle (5 l) and transferred anoxically into sterile serum bottles (160 ml) with a gas tight outlet tubing as described above. Bottles were closed with butyl stoppers (Supelco) and crimp seals and kept in the dark at 4°C for no more than 3 h until further processing.

### Nutrient and metal analyses

Nitrite, ammonium, and sulfide were analyzed photometrically according to Griess (1879), Krom (1980), and Cline (1969), respectively. Along with nitrate and phosphate, nitrite concentrations were additionally determined by flow-injection analysis (FIA; SAN++, Skalar). Sulfate was measured by ion chromatography (882 Compact IC plus, Metrohm).

Inductively coupled plasma-mass spectrometry (ICP-MS; Element2, Thermo-Fisher) was used to measure concentrations of total and dissolved metal fractions. Additionally, Fe(II)/(III) concentrations were determined photometrically in both filtered and unfiltered samples with the ferrozine assay (Stookey, 1970). Fe(II) was measured directly and Fe(II)+Fe(III) was determined after reduction with hydroxylamine hydrochloride (Viollier et al., 2000).

### Methane, in/organic carbon, and stable carbon isotopes

For dissolved methane concentration measurements, a 20 ml N_2_ headspace was introduced and exchanged for sample water. After overnight equilibration, the gas phase was analyzed with a gas chromatograph (GC; Agilent 6890N, Agilent Technologies) equipped with a Carboxen 1010 column (30 m × 0.53 mm, Supelco) and a flame ionization detector (FID). Solubility constants were used to calculate the original amount of CH_4_ in the water phase (Wiesenburg and Guinasso, 1979). To analyze the ^13^C/^12^C isotopic ratios of the headspace methane, injected samples were first purified and concentrated in a trace gas unit (T/GAS PRECON, Micromass UK Ltd.) by a series of chemical (magnesium perchlorate, Carbo-Sorb and Sofnocat) and cold (liquid N_2_) traps. Subsequently, the purified gas was transferred to a connected isotope ratio mass spectrometer (IRMS; GV Instruments, Isoprime). Results are expressed in the conventional δ^13^C-notation, normalized to the Vienna Pee Dee Belemnite (VPDB) reference standard. The reproducibility of the method based on replicate standard measurements was generally better than 1.4‰.

A total carbon analyzer (TOC-L, Schimadzu) was used to quantify DIC and DOC. Filtered water samples were injected directly (for total dissolved C determination) or after acidification with HCl (20 mM final concentration; for DOC determination) and measured with a non-dispersive infrared detector (NDIR) after volatilization to CO_2_. Dissolved inorganic C (DIC) was quantified as the difference between dissolved total and dissolved organic C. To determine the carbon isotopic composition of DIC, 1 ml of the remaining water sample was immediately introduced into a He-filled 3.7 ml Exetainer (Labco Ltd). Following acidification (100 μl 85% H_3_PO_4_) and overnight equilibration, released CO_2_ was analyzed in the headspace with a preparation system (MultiFlow, Isoprime) coupled to an IRMS (Micromass, Isoprime). δ^13^C-DIC values are also reported relative to VPDB, with a reproducibility of 0.1‰.

Based on the CH_4_ concentration profile and corresponding δ^13^C change, a zone of active methane oxidation was identified. A fractionation factor (α_*c*_) for methane oxidation was then calculated as follows (Whiticar and Faber, 1986):

$$\begin{array}{l} \delta^{13}C\;\left(‰\right)=\left[\delta^{13}C_{0}+1000\;\cdot \;f^{\left(\frac{1}{\alpha_{c}}-1\right)}\right]-1000 \end{array}$$

where δ^13^*C*_0_ represents the C isotopic composition of the methanogenic source (measured in near-bottom waters) and δ^13^*C* is the C isotopic composition of methane at various depths in the zone of methane oxidation. *F* represents the fraction of residual methane relative to the concentration in near-bottom waters.

### Flux calculations

Fluxes of solutes in the water column toward and within the redox transition zone were calculated according to:

$$\begin{array}{l} J=-K_{z}\frac{\partial C}{\partial X} \end{array}$$

where *K*_*z*_ is the vertical turbulent dispersion coefficient and $\frac{\partial C}{\partial X}$ is the concentration change over the corresponding depth range. A low diffusion coefficient of 4·10^−3^ cm^2^ s^−1^ was chosen (Walter et al., 2014). Gradients were estimated by applying a linear regression over the depth interval, where concentration profiles showed the steepest slope. The resulting diffusive fluxes were converted to electron equivalents (e^−^) by multiplying them with the electron accepting/donating capacity of the respective species.

### Catalyzed reporter deposition-fluorescence *in situ* hybridization

Formaldehyde-fixed (2% [v/v] final concentration) water samples (corresponding to the incubation depths) were incubated for 30 min at room temperature (RT) before being filtered onto 0.2 μm polycarbonate filters (GTTP, Millipore). Filters were rinsed with 1x phosphate buffered saline solution (PBS) and stored at −20°C until further handling. Standard CARD-FISH (Pernthaler et al., 2002) was performed with horseradish peroxidase-labeled oligonucleotide probes (purchased from Biomers) binding to specific 16S rRNA gene sequences of targeted microbial groups (Supplementary Table 1). In short, bacterial cells were permeabilized with lysozyme (10 mg ml^−1^, 1 h at 37°C). Archaeal cell walls were permeabilized with proteinase K (15 μg ml^−1^, 3 min at RT) for probes AAA-FW-641, -834, and ANME-1-350 or with sodium dodecyl sulfate (SDS; 0.5% [v/v], 10 min at RT) for probe ANME-2-538. Subsequently, endogenous peroxidase activity was inhibited (0.1 M HCl, 10 min at RT) followed by hybridization (2.5 h at 46°C) and tyramide signal amplification (Oregon Green 488, 1 μl ml^−1^, 30 min at 37°C). Finally, filter pieces were counterstained with 4′,6-diamidino-2-phenylindole (DAPI; 1 μg ml^−1^, 5 min at RT), embedded in Citifluor/Vectashield (4:1) and mounted onto glass objective slides. Total cells stained with DAPI and cells belonging to microbial groups targeted with CARD-FISH were enumerated with a grid ocular of an epifluorescence microscope (Axioskop 2, Zeiss) by counting 20 randomly selected fields of view. Total cell numbers were quantified by DAPI staining and fractions of the different groups were calculated. Probes Mgamma84 and −705 and AAA-FW-641 and −834 were used in a 1:1 mix. Probe NON338 served as a negative control for the procedure.

### DNA extraction, polymerase chain reaction and cloning

DNA for phylogenetic analysis was collected by filtering at least 150 ml water through polycarbonate Nuclepore Track-Etched Membrane filters (0.2 μm pore size; Whatman). Filters were frozen immediately and stored at −70°C until DNA was extracted using a Fast DNA Spin Kit for Soil (MP Biomedicals). The primers A189F (5′-GGNGACTGGGACTTCTGG-3′) (Holmes et al., 1995) and mb661R (5′-CCGGMGCAACGTCYTTACC-3′) (Costello and Lidstrom, 1999) were used to target subunit A of the pMMO gene (*pmoA*). To target the 16S rRNA of NC 10 bacteria, primers NC10-202Fdeg (5′-RACCAAAGGRGGCGAGCG-3′) and NC10-1043Rdeg (5′-TCTCCRCGYTCCCTTGCG-3′) were applied (Deutzmann and Schink, 2011). PCR was done using the GoTaq Flexi DNA Polymerase kit (Promega), in 20 μL assays containing 2 μL undiluted template DNA, 1x amplification buffer, 2.5 mM MgCl_2_, 0.2 mM dNTPs, 0.3 μM of each primer, and 1.25 U Taq polymerase. PCR conditions for *pmoA* detection were as follows: initial denaturation at 94°C for 2 min, then 30 cycles of 1 min denaturation at 94°C, 1 min primer annealing at 60°C, and 1 min elongation at 72°C, followed by a final elongation step of 10 min at 72°C. PCR conditions targeting the 16S rRNA of NC 10 bacteria were set according to Deutzmann and Schink (2011). Positive PCR products of three separate 50 μl reactions were combined and concentrated to 25 μl using a Wizard SV PCR cleanup kit (Promega). Amplicon length was checked on a 1.3% agarose gel after Midori Green staining and blue light illumination.

Clone libraries were constructed using the pGEM-T and pGEM-T Easy Vector Systems Cloning Kit (Promega). Fifty white clones of each transformation culture were transferred to a fresh LB plate and grown overnight at 30°C. The primers SP6 (5′-ATTTAGGTGACACTATAG-3′) and T7 (5′-TAATACGACTCACTATAGGG-3′) were used to check for correctly sized inserts by performing a colony PCR using the reagents supplied by the Kapa2G Robust PCR kit (Kapa Biosystems) in 25 μL mixtures containing: 5x buffer B, 0.2 mM dNTPs, 0.2 μM of each primer, and 0.5 U Kapa2G Robust polymerase and a small amount of biomass. PCR conditions were set as follows: initial denaturation at 95°C for 3 min, then 30 cycles of 15 s denaturation at 95°C, 15 s annealing at 95°C, and 30 s extension at 55°C, followed by a final extension step of 1 min at 72°C. Amplicon length was checked on a 1.3% agarose gel. PCR products with the expected length were purified using the Wizard SV PCR cleanup kit (Promega) and sent for Sanger sequencing to Eurofins Genomics (Cologne, Germany).

The obtained sequences were aligned using the Muscle implementation of MEGA6 (Tamura et al., 2013). After trimming and visual inspection of the alignment and exclusion of sequences with obvious sequencing or PCR amplification errors, the evolutionary history was inferred by the Neighbor-Joining approach (Saitou and Nei, 1987) using Jukes Cantor correction (Jukes and Cantor, 1969) and pairwise deletion handling of gaps. Reported sequences are deposited in the European Nucleotide Archive under the accession numbers LT617822-881.

### Methane oxidation potential

Incubation experiments to quantify the methane oxidation potential in the water column were carried out shortly after sampling (~3 h later) with water collected in 160 ml serum bottles. Depths were chosen based on the different redox regimes: 12 m (oxic), 14 m (oxycline), 15 m (suboxic), and 16 and 18 m (anoxic). Incubations were prepared analogous to the procedure described in Holtappels et al. (2011) for ^15^N-labeling experiments. First, water was degassed with He for 10–15 min to remove traces of contaminant O_2_, as well as background CH_4_. Except for two non-amended bottles (dark and light setup), all individual bottles received additions of electron acceptors (Table 1). Electron-acceptor solutions were prepared with sterile (autoclaved) anoxic (boiled and cooled under N_2_) Nanopure water. The O_2_ solution was prepared by letting Nanopure water equilibrate with the atmosphere, followed by sterilization (autoclaving the sealed and crimped bottle). Subsequently, all bottles were supplemented (~5 ml) with a saturated ^13^CH_4_ (99 at.%, Campro Scientific) solution (anoxic and sterile) resulting in a final CH_4_ concentration of about 50 μM in each bottle. After the CH_4_ addition, water was immediately distributed into 12 ml Exetainers without a headspace, preventing air contact by allowing the flow of He to push the water out (Holtappels et al., 2011). Exetainers were incubated at *in situ* temperatures (6°C) under dark or light (~5 μE m^−2^ s^−1^) conditions (Table 1) and sampled destructively by terminating microbial activity with the addition of 200 μl ZnCl_2_ (50% [w/v]) after 0, 6, 12, 24, and 48 h. Exetainers were stored upside down at RT until analysis, which was performed by GC-IRMS as described above for the determination of δ^13^C-DIC. CH_4_ oxidation was measured as the production of ^13^C-DIC as published previously (Oswald et al., 2015, 2016). Briefly, δ-values of the DIC were first converted to a fractional abundance of ^13^C based on the absolute abundance ratio of ^13^C/^12^C (0.0111796) (Coplen et al., 2002). Using the ambient DIC concentration (~7.4 mM), absolute ^13^C-DIC concentrations were then determined. The temporal change in ^13^C (reflecting the production of MO derived ^45^CO_2_) between 0 h and the different time points was used to calculate methane oxidation rates by linear regression over the incubation time. To compare all experiments, maximum potential rates were calculated during the initial ~12 h linear segment. At least in samples from the oxycline and above, we added ^13^CH_4_ at concentrations much higher than the ambient concentration. Similarly, all other solutes were added in excess of their natural occurrence at the sampled depths, hence, estimated rates must be considered as potential methane oxidation.

**Table 1:** **Setups prepared for quantifying methane oxidation potential**.

**Setup**	**Stock**	**Treatment (μM)**	**Conditions**	**Depths (m)**
Dark	–	–	dark	12, 14, 15, 16, 18
Light	–	–	light	14, 15, 16
Oxygen	saturated O_2_	15	dark	14, 15, 16
Nitrate	100 at.% ^15^${\text{NO}}_{3}^{-}$	40	dark	14, 15, 16
Nitrite	100 at.% ^15^${\text{NO}}_{2}^{-}$	20	dark	14, 15, 16
Iron(III)	ferrihydrite suspension^a^	100	dark	14, 15, 16
Manganese(IV)	birnessite suspension^b^	100	dark	14, 15, 16

a *Ferrihydrite was synthesized according to Cornell and Schwertmann (2006)*.

b *Birnessite was synthesized according to Golden et al. (1987)*.

Testing potential links between MO and denitrification, we also determined NO_*x*_ consumption and N_2_ production by denitrification (and/or anaerobic ammonium oxidation; anammox) in incubations spiked with ^15^${\text{NO}}_{3}^{-}$ or ^15^${\text{NO}}_{2}^{-}$. Concentrations of ${\text{NO}}_{3}^{-}$ and ${\text{NO}}_{2}^{-}$ were quantified by FIA (see above). After generation of an He headspace (2 ml) and overnight equilibration, headspace concentrations of ^30^N_2_ (and ^29^N_2_) were measured by IRMS (Delta-V Advantage IRMS, Thermo Scientific), calculated from the integrated peak areas in relation to an air standard (Holtappels et al., 2011).

In incubations with added Fe(III) or Mn(IV), additional samples were taken to quantify dissolved/reduced (<0.45 μm, cellulose acetate filter) metal concentrations. These were directly filtered into vials containing either HCl (0.5 M final concentration) or HNO_3_ (0.1 M final concentration) to preserve Fe and Mn, respectively. Dissolved Fe(II)/(III) was determined with the ferrozine assay and Mn was determined by ICP-MS as described above.

## Results

### Geochemical conditions in the Lake La Cruz water column

**Figure 1:**
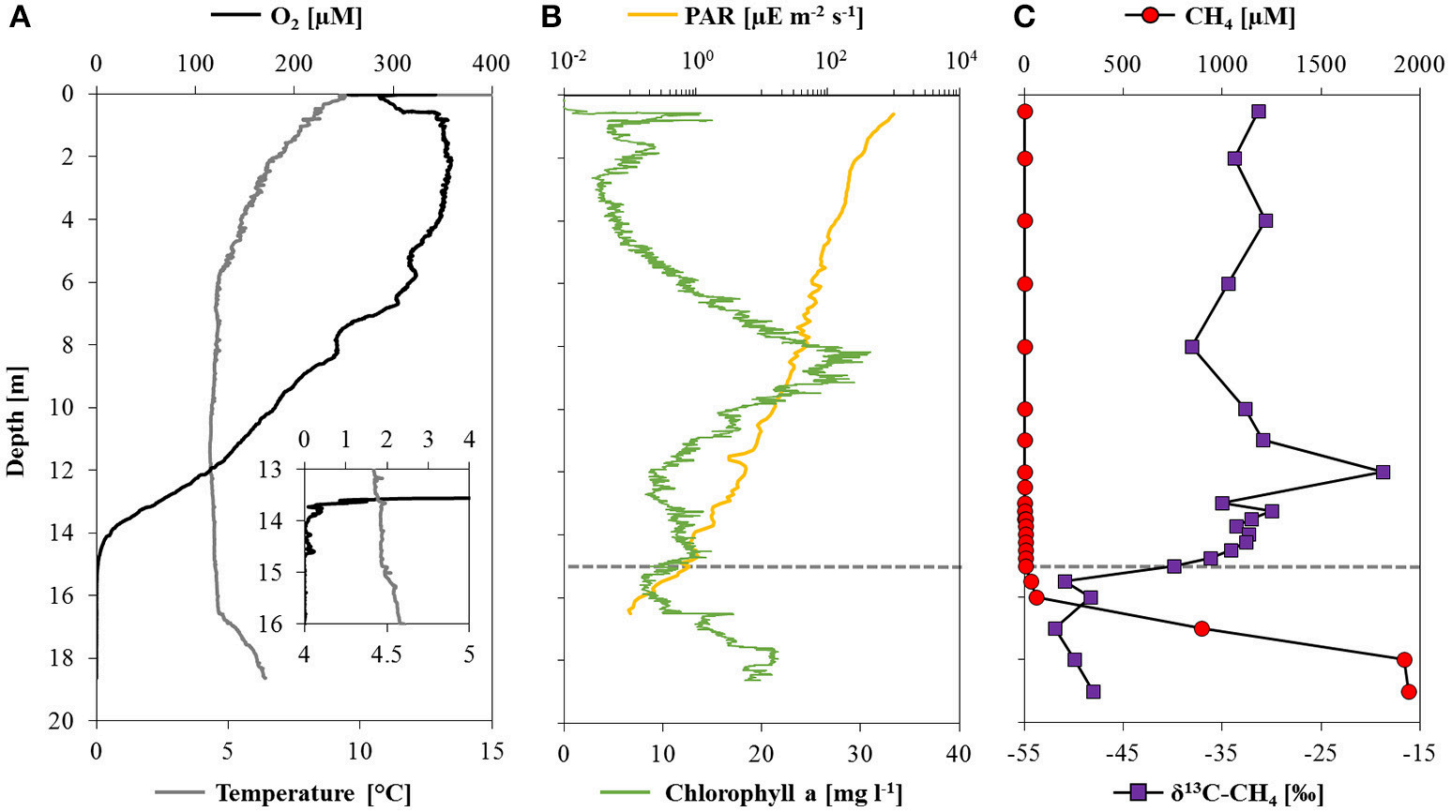
**Physicochemical parameters, methane concentrations and stable carbon isotope ratios in Lake La Cruz. (A)** Profiles of oxygen and temperature with a detailed view from 13 to 16 m depth, where the oxygen concentration was measured with a trace optode; **(B)** profiles of photosynthetically active radiation (note the logarithmic scale) and chlorophyll a concentrations; **(C)** depth profiles of methane concentrations and the corresponding δ^13^C-CH_4_. The dashed line denotes the location of the oxycline.

Oxygen concentrations were about 260 μM in surface waters, reaching a maximum concentration of 360 μM at depths between 2 and 4 m (Figure 1A). Thereafter O_2_ decreased gradually with the strongest O_2_ gradient (oxycline) between 13 and 14 m, and complete O_2_ depletion (i.e., <20 nM O_2_) at 14.6 m. Rising temperatures and drastic changes in pH and conductivity below 16.5 m (Supplementary Figure 1A) indicated the location of the thermo- and chemocline, respectively. Relative to the total irradiation at the water surface (~1200 μE m^−2^ s^−1^) PAR decreased to 0.14% (1.9 μE m^−2^ s^−1^) at the oxycline, and could be detected at a maximum depth of 16.5 m (0.1 μE m^−2^ s^−1^; Figure 1B). Chlorophyll a was present throughout the water column with a maximum of 30 mg l^−1^ at 8.5 m.

Methane concentrations were highest above the sediment-water interface (2.2 mM) and decreased stepwise in the hypolimnion: first very sharply toward the chemocline at 16.5 m (~50 μM), and a second time with a less pronounced concentration gradient within the oxycline, where it was almost completely consumed (Figure 1C). Within the oxic epilimnion, CH_4_ concentrations varied between 0.1 and 4.6 μM, which is well above the detection limit (~0.01 μM). The δ^13^C-CH_4_ was relatively stable below the chemocline with values around −50‰, and increased significantly from −49 to −28‰ between 16 and 12 m. In oxic waters, δ^13^C-CH_4_ shifted toward lower values again (~−33‰). Based on both CH_4_ concentration gradients and isotopic signatures, a zone of active methane oxidation could be defined between 16 and 12 m. The apparent (community) fractionation factor for MO calculated across this zone using a closed-system Rayleigh model approach was −1.005.

**Figure 2:**
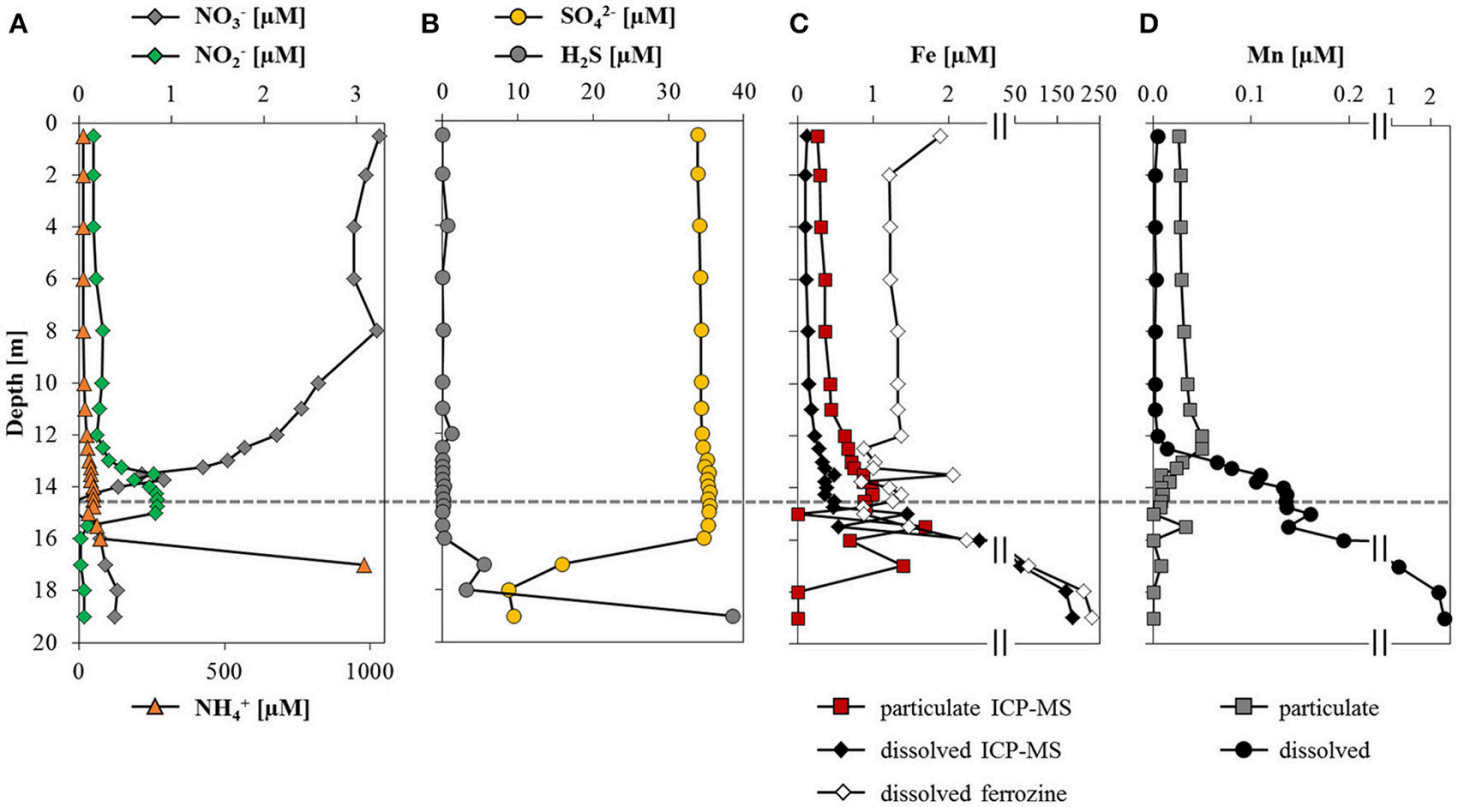
**Water column distribution of dissolved ions and metal species**. Depth profiles of **(A)** nitrate, nitrite and ammonium concentrations; **(B)** sulfate and total sulfide concentrations; **(C)** dissolved and particulate iron concentrations quantified by ICP-MS along with dissolved Fe(II) determined with the ferrozine assay; and **(D)** dissolved and particulate manganese as determined by ICP-MS. The dashed line represents the depth of the oxycline. Note the break in the x-axis in **(C,D)**.

Nitrate concentrations fluctuated around 3 μM in the oxic zone and decreased sharply at the oxycline to below 0.4 μM (Figure 2A). Ammonium concentrations were ~12 μM in the oxic epilimnion and increased steadily to 70 μM at 16 m. Below this depth, a sharp ammonium gradient extended to the sediment surface where concentrations were in the mM range (1 mM at 17 m, values below this depth are not displayed as they were outside the calibration). Nitrite was only detected between 13 and 16 m depth, with a maximum of 0.8 μM at 14.5 m. Sulfate concentrations were stable around 35 μM in the epilimnion down to 16.5 m, from where concentrations decreased sharply to 8 μM in near-bottom waters (Figure 2B). Sulfide reached values >35 μM near the sediment surface but decreased to detection limit above 16 m.

Total iron concentrations were highest (~200 μM) above the sediment-water interface, and decreased toward the oxycline, above which measured Fe concentrations were below 0.5 μM (Figure 2C). The discrepancy between dissolved Fe determined photometrically and by ICP-MS above the oxycline is probably due to measuring near the detection limit with the ferrozine assay (~0.5 μM). Above 16.5 m, the particulate iron fraction accounted for about 70% of total iron, whereas the dissolved fraction was predominant at depth. The ferrozine assay confirmed that below the oxycline the majority of dissolved iron was present as reduced Fe(II) and particulate iron was mainly in the form of Fe(III). In the oxic zone, only particulate (oxidized) manganese was detected (0.04 μM; Figure 2D), in contrast to anoxic waters, where manganese was almost exclusively present in its dissolved (reduced) state. The highest manganese concentrations (2.3 μM) were above the sediment-water interface.

Our flux calculations of e^−^ equivalents revealed that oxygen was the dominant electron acceptor in the water column of Lake La Cruz. The main electron donors apart from organic matter (DOC max. 80 μM at 4 m; Supplementary Figure 1C) were ammonium and methane. The calculated downward e^−^ flux from O_2_ was 7.3 ± 0.03 mmol e^−^ m^−2^ d^−1^ and the upward e^−^ flux from CH_4_ was −11.1 ± 2.0 mmol e^−^ m^−2^ d^−1^. Electron fluxes of the other species were considerably lower, ranging between 0.01 ± 0.09 mmol e^−^ m^−2^ d^−1^ for ${\text{SO}}_{4}^{2-}$, −1.7 ± 0.45 mmol e^−^ m^−2^ d^−1^ for ${\text{NH}}_{4}^{+}$ and −0.03 ± 0.01 mmol e^−^ m^−2^ d^−1^ for Fe(II). Total sulfide and manganese fluxes were negligible with regards to the overall electron balance (Table 2). The sum of the different electron fluxes suggests an electron acceptor deficit on the order of 5.3 ± 2.0 mmol e^−^ m^−2^ d^−1^, implying that a relatively large portion of the total upward flux of methane from the methanogenic zone is not accounted for by oxidation with O_2_ from the upper water column.

**Table 2:** **Calculated electron fluxes across the zone of methane oxidation**.

**Electron acceptors**		**Electron donors**	
**Species**	**J (mmol e^−^m^−2^ d^−1^)**	**Species**	**J (mmol e^−^m^−2^ d^−1^)**
O_2_	7.3±0.03	CH_4_	−11.1±2.0
${\text{NO}}_{3}^{-}$	0.15±0.02	Fe^2+^	−0.03±0.01
${\text{SO}}_{4}^{2-}$	0.01±0.09	${\text{NH}}_{4}^{+}$	−1.7±0.45
Sum	7.5±0.04	Sum	−12.8±2.04

### Microbial community structure

**Figure 3:**
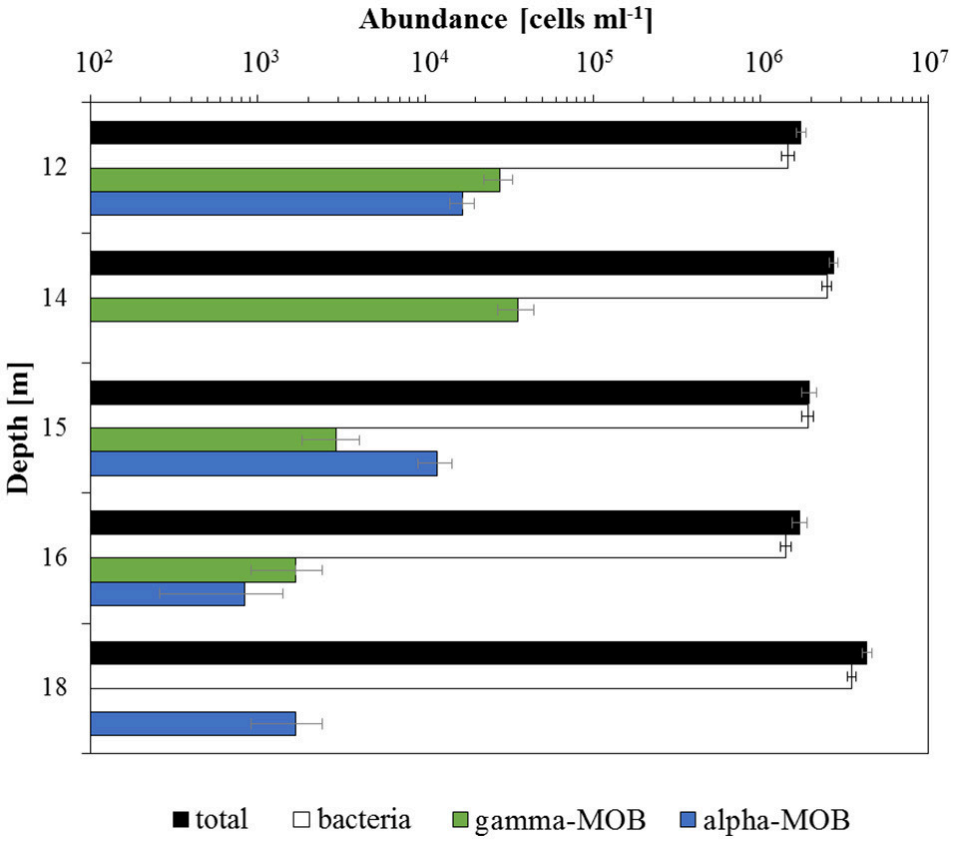
*****In situ*** cell abundances of different microbial groups**. Total cell numbers determined by DAPI cell counting. Cell abundances of bacteria (probes EUB338 I-III), gamma-MOB (probe mix Mgamma84+705), and alpha-MOB (probe Ma450) were assessed using CARD-FISH. Note the logarithmic scale. Error bars denote the standard error of the mean between counted fields of view (20).

Total cell density (as determined by DAPI counts) was rather constant at 12, 14, 15, and 16 m, ranging between 1.7·10^6^ cells ml^−1^ (16 m) and 2.7·10^6^ cells ml^−1^ (14 m; Figure 3). Significantly higher total cell numbers were detected at 18 m depth (4.3·10^6^ cells ml^−1^). Similar to total cell numbers, bacterial cell abundance (probes EUB338 I-III) showed no clear depth trend, but again highest numbers were determined for 18 m (3.5·10^6^ cells ml^−1^) and the lowest cell abundance was observed at 16 m (1.4·10^6^ cells ml^−1^). As an approximation, we can roughly estimate archaeal cell abundance as the difference in total and bacterial counts. Based on this difference, it appears that archaea were at least one order of magnitude less abundant than bacteria (and two orders of magnitude lower at 15 m). Aerobic MOB were detected at all sampled depths. Gamma-MOB (probe mix Mgamma84+705) cell numbers were highest at 14 m with 3.6·10^4^ cells ml^−1^ (1.3% of total DAPI counts), and decreased with depth; they were not detected at 18 m. Alpha-MOB (probe Ma450) were most prominent at 12 m (1.7·10^4^ cells ml^−1^, 1% of total DAPI counts). Although completely absent at 14 m, alpha-MOB were detected at all other depths, amounting to 1.7·10^3^ cells ml^−1^ at 18 m (0.04% of total DAPI counts). In relative terms, gamma-MOB were dominant at 12, 14, and 16 m, whereas alpha-MOB were more numerous at 15 and 18 m. Known groups of ANME belonging to ANME-1 (probe ANME-1-350), ANME-2 (probe ANME-2-538), and AOM-associated archaea (AAA; probe mix AAA-FW-641+834) were not detected. Probe NON338 did not yield any hybridization signals, confirming that there was no background interference.

**Figure 4:**
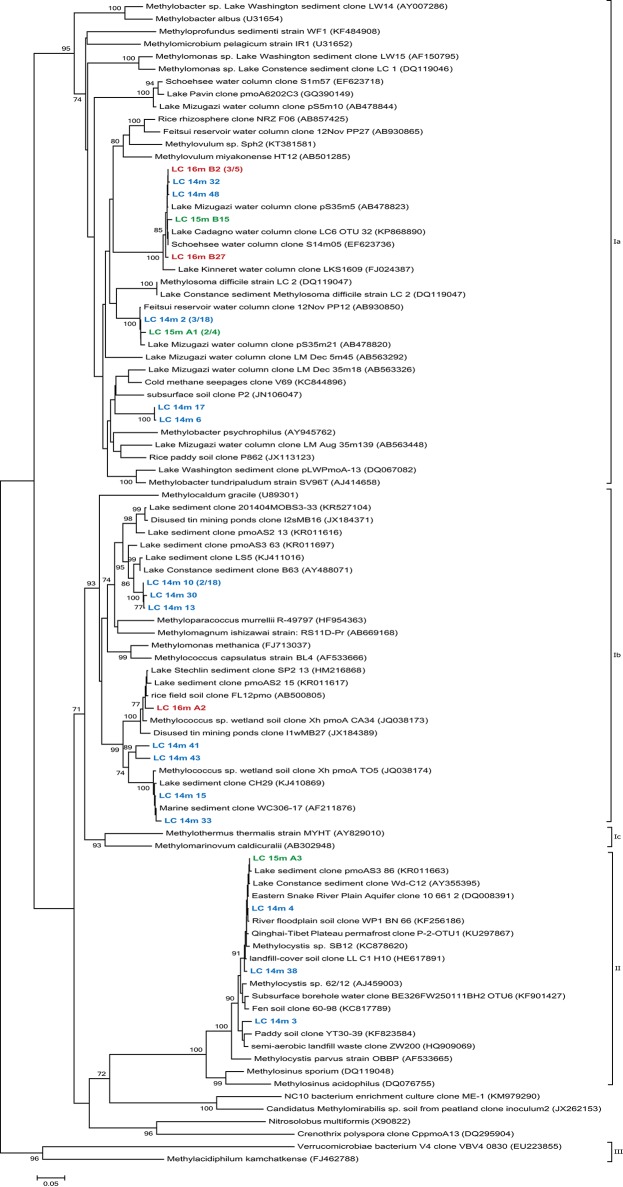
**Phylogenetic tree of ***pmoA*** gene sequences from the La Cruz water column at and below the oxycline, 14m (LC 14m), 15m (LC 15m), and 16m (LC 16m) depth**. The tree was constructed using the Neighbor-Joining method, based on alignment of 571 nucleotide positions with the Jukes Cantor correction, choosing the partial deletion option. Analyzed clones are colored according to sampling depth and bootstrap values >70% (*n* = 1000) are displayed at the branch nodes. The scale bar denotes the number of changes per nucleotide position.

**Figure 5:**
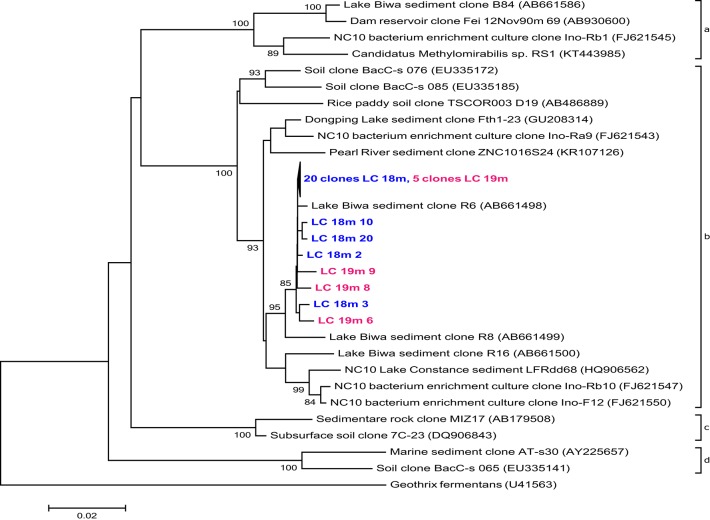
**Phylogenetic assignment of 16S rRNA gene sequences within the phylum NC 10 retrieved from the La Cruz water column, 18m (LC 18m) and 19m (LC 19m) depth**. The tree was constructed using the Neighbor-Joining method, based on alignment of 823 nucleotide positions, using Jukes Cantor correction and partial deletion gap handling. Bootstrap values >70% (1000 resamplings) and a scale bar indicating the number of changes per nucleotide position are shown. The sequence of *Geothrix fermentans* was included as outgroup.

*PmoA* sequences recovered from 14, 15, and 16 m predominantly fell within the type I (gamma-) MOB and formed four main clusters (Figure 4). The first three were most closely related to uncultured species from other freshwater lakes, reservoirs, and sediments: Lake Mizugaki (Kojima et al., 2009; Tsutsumi et al., 2011), Feitsui Reservoir (Kojima et al., 2014), Lake Cadagno (Milucka et al., 2015), Lake Kinneret (Junier et al., 2010), Lake Schoehsee (unpublished), Lake Constance (Pester et al., 2004), and lakes on the Yunnan Plateau (Liu et al., 2015). The fourth cluster was closely related to an uncultured *Methylococcus* species from a wetland in northeast China (Yun et al., 2013) and an uncultured bacterium from lake sediments (Liu et al., 2015). Sequenced clones belonging to type II (alpha-) MOB were most closely related to cultured and uncultured species of *Methylocystis*. Sequences associated with *pmoA* of *M. oxyfera* (NC 10 phylum) or *Crenothrix polyspora* were not found at 14, 15, or 16 m depth. However, 16S rRNA gene sequences belonging to the NC 10 phylum were recovered from 18 to 19 m depth (but not from any other depth; Figure 5), and were most closely related to uncultured species from Lake Biwa sediments (Kojima et al., 2012) but not directly to known nitrate-reducing methanotrophs, such as *M. oxyfera*.

### Methane oxidation potential

**Figure 6:**
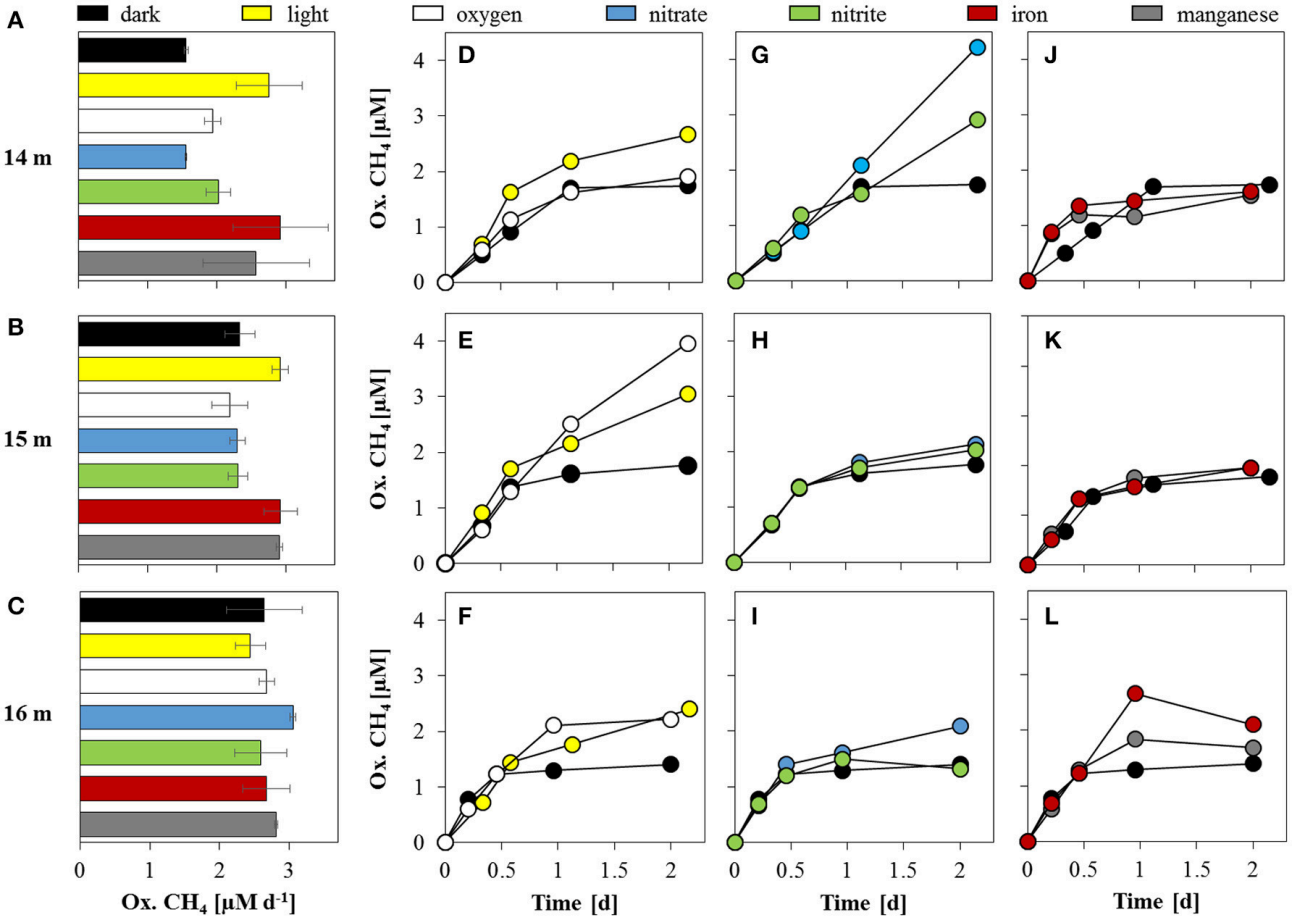
**Methane oxidation potential at and below the oxycline**. Methane oxidation rates **(A–C)** and corresponding oxidation time series are displayed for 14 m (top panel), 15 m (center panel), and 16 m (bottom panel). **(D–F)** Time series of oxidized methane under dark and light conditions; **(G–I)** with additions of nitrate and nitrite; and **(J–L)** iron and manganese. The dark control series is displayed for reference in all panels. Methane oxidation rates in **(A–C)** are calculated based on the initial ~12 h linear segments of the time series in **(D–L)**.

Incubation experiments to quantify methane oxidation potential and dynamics were conducted with water collected from four different zones: under oxic conditions (~100 μM O_2_), within the oxycline (60 nM O_2_), under suboxic conditions (<20 nM O_2_) and under true anoxia/euxinia. The effects of a variety of added electron acceptors [O_2_, ${\text{NO}}_{3}^{-}$, ${\text{NO}}_{2}^{-}$, Fe(III), and Mn(IV)] and light on methane oxidation were tested separately and compared to the control with only added ^13^CH_4_. In these dark control incubations, MO potential increased with depth from 12 m (0.7 ± 0.02 μM d^−1^; Supplementary Figure 2A) to 16 m (2.6 ± 0.55 μM d^−1^; Figures 6A–C). At 18 m, rates were notably lower with 0.1 ± 0.03 μM d^−1^ (Supplementary Figure 2A). In general, methane oxidation was non-linear over time; after the initial 12–24 h, oxidation either slowed or ceased (Figures 6D–F), suggesting the onset of substrate limitation at some point during the incubations. Only at 12 m methane oxidation was constant over the entire course of the experiment (Supplementary Figure 2B).

The addition of 15 μM oxygen (at 14, 15, and 16 m) led to initial methane oxidation rates similar to the corresponding dark controls (Figures 6A–C), but caused an approximate doubling of the total methane turnover at both 15 m (4.0 μM) and 16 m (2.2 μM; Figures 6E,F) at the end of the incubation period. The oxygen supplement resulted in almost constant CH_4_ oxidation (i.e., linear increase of the CH_4_-derived CO_2_) throughout the experiment at 15 m (Figure 6E). The addition of O_2_ appeared to have no effect on concentration-time curves only at 14 m (Figure 6D).

The MO potential was enhanced under light conditions at 14 m (2.8 ± 0.47 μM d^−1^) and 15 m (2.9 ± 0.11 μM d^−1^), whereas MO potential at 16 m (2.4 ± 0.21 μM d^−1^) was slightly lower than the dark control (Figures 6A–C). In contrast to the dark controls, methane oxidation never reached a plateau in any of the light incubations (Figures 6D–F). Although MO slowed after ~12 h, a higher total methane turnover was observed under light conditions at all depths, with a maximum of 3.1 μM (over the 2-day total incubation period) at 15 m.

Relative to the dark control experiments, the addition of nitrate or nitrite did not seem to have any noticeable effect on the MO potential. Yet interestingly, and in contrast to the incubations at 15 and 16 m, at 14 m, the addition of NO_x_ led to an almost linear increase in oxidized CH_4_ over the entire incubation period, and very high total CH_4_ turnover (4.2 μM with ${\text{NO}}_{3}^{-}$ and 2.9 μM with ${\text{NO}}_{2}^{-}$; Figure 6G), suggesting that substrate limitation was mitigated by the supplemented NO_x_. The addition of ${\text{NO}}_{3}^{-}$ induced the greatest increase in net CH_4_ oxidation at 14 m compared to all other substrate additions and depths. Total methane turnover was only slightly enhanced by the NO_x_ amendment at 15 and 16 m.

Methane oxidation potential and oxidation kinetics were very similar with additions of both iron(III) (ferrihydrite) and manganese(IV) (birnessite). Maximum MO potential was twice as high as in the dark control at 14 m, or 2.9 ± 0.68 and 2.6 ± 0.77 μM d^−1^ with the addition of Fe(III) and Mn(IV), respectively (Figure 6A). In all setups with added Fe(III)/Mn(IV), MO stagnated after the initial 12 h and net oxidized CH_4_ at the end of the incubation period was within the same range as observed in the dark incubation (Figures 6J–L). The highest final CH_4_ turnover in the Fe(III) and Mn(IV) setups was 2.1 μM (16 m) and 2.0 μM (15 m), respectively.

## Discussion

### Redox regimes in Lake La Cruz

Primary production via photosynthesis took place in surface waters with maximum activity around 8.5 m, corresponding to a peak in chlorophyll a (Figure 1B). Below the sunlit zone, organic matter respiration was evident from the incremental depletion of available electron acceptors more or less following the canonical redox cascade. The O_2_ concentration profile outlines a zone of net O_2_ consumption between 13.5 and 14 m (defined here as the oxycline; dashed line in Figure 1). Below the oxycline, the decrease in ${\text{NO}}_{3}^{-}$ and a concurrent ${\text{NO}}_{2}^{-}$ maximum (14–15 m; Figure 2A), provides evidence for an active zone of denitrification. Finally, an increase in dissolved/reduced Mn (below 12 m) and reduced Fe (below 16 m) indicates ongoing metal reduction in the hypolimnion (Figures 2C,D). As indicated by the concomitant decrease of ${\text{SO}}_{4}^{2-}$ and the increase in H_2_S toward the sediment, sulfate reduction may occur below 17 m in the water column (Figure 2B).

### Methane sources and sinks

Based on methane concentration profiles, corresponding isotopic signatures, and measured methane oxidation potential we identified four distinct zones: the methane production/methanogenic zone (>19 m), the (non-reactive) methane diffusion zone (19–16 m), the methane oxidation zone (16–12 m), and the oxic zone (<12 m).

#### Methane production/methanogenic zone

Although we did not analyze CH_4_ concentrations and isotopic composition in the sediments, maximum water column CH_4_ concentrations were nearest to the sediment (Figure 1C) suggesting that it is produced within the sediments and diffuses into the water column. The δ^13^C-CH_4_ in the deepest methane sample was about −50‰, which falls between the range of ratios indicative for biogenic (<−60‰) and thermogenic origin (>−50‰) (Whiticar, 1999). However, the isotopic discrimination factor between CH_4_ and DIC (Δδ^13^C_CH4−DIC_ = 51‰) supports a biotic (i.e., methanogenesis in the sediment) rather than a thermogenic source (Whiticar, 1999). Further analysis of H isotopes of methane (Stolper et al., 2015) could confirm this indication. The comparatively high δ^13^C values for the near-sediment CH_4_ may indicate partial consumption and C isotope fractionation by AOM within the sediments.

#### Methane diffusion zone

A sharp decrease in methane concentrations was measured between the sediment surface and 16 m, yet the δ^13^C-CH_4_ was more or less invariant (~−50‰) throughout this part of the water column (Figure 1C). The constant δ^13^C-CH_4_ suggests the absence of active MO in this zone, as methanotrophs would preferentially incorporate the light carbon isotope, ^12^C, leaving the residual pool of CH_4_ enriched in ^13^C. The measured oxidation potential at 18 m confirms that MO in this zone was minor (Supplementary Figure 2). Therefore, this initial steep gradient appears to be a result of diffusive mixing and transport of sediment-derived methane into the deep water column and toward the reactive methane oxidation zone several meters above.

#### Methane oxidation zone

A subtle convex CH_4_ profile was visible between 16 and 12 m. This decrease coincided with a shift to notably higher δ^13^C-CH_4_ values (−48‰ at 16 m to −19‰ at 12 m; Figure 1C), which, along with measured MO rates at depths throughout this zone (Figure 6), confirms ongoing microbial methane consumption, fractionating the stable C isotopes. While turbulent diffusive exchange between the reaction zone and the non-reactive zone below can already cause δ^13^C gradients in the water column where MO is absent (analogously to what is observed for other substrates, e.g., Lehmann et al., 2007), the observed profiles suggest that reaction must occur below 14 m. The calculated fractionation factor of −1.005 is relatively low compared to typical values reported in the literature, but is not inconsistent with aerobic MO as the dominant methane removal mechanism (Templeton et al., 2006). Yet, considering (i) that the range of reported C isotope fractionation factors for aerobic MO and sulfate-dependent AOM overlap to some degree (Templeton et al., 2006; Holler et al., 2009; Rasigraf et al., 2012), (ii) that the C isotope effects for other processes that may be relevant in Lake La Cruz are unknown (e.g., ${\text{NO}}_{3}^{-}$/${\text{NO}}_{2}^{-}$/Fe/Mn-coupled AOM), and (iii) that, independent of the mode of MO, the microbe-level isotope effects may be under-expressed at the ecosystem level (i.e., Blees et al., 2014), we cannot unambiguously confirm aerobic MO based on the isotope data alone.

#### Oxic zone

In the oxic zone, methane concentrations were very low (≤ 300 nM) compared to the deep water, indicating that most methane was consumed before reaching the epilimnion. These concentrations were likely too low to sustain significant MO there. Indeed, the MO potential above the oxycline (12 m; Supplementary Figure 2) was an order of magnitude lower than below. The oxygenated region of the water column is likely unfavorable even for aerobic methanotrophs, not only because of methane limitation, but likely also due to the inhibitory effects of high O_2_ concentrations (Rudd and Hamilton, 1975) and light intensity (Dumestre et al., 1999; Murase and Sugimoto, 2005). Interestingly, the δ^13^C-CH_4_ decreased again (−35‰) in the epilimnion above the methane oxidation zone (Figure 1). This comparatively ^13^C-depleted isotopic signature could be explained by mixing of atmospheric methane (−47‰) (Wuebbles and Hayhoe, 2002) and methane from the oxidation zone. Alternatively, lateral sources of CH_4_ from the littoral zone (Juutinen et al., 2003; Murase et al., 2005) or water-column methanogenesis (Grossart et al., 2011; Bogard et al., 2014) may produce this C isotopic shift in the oxic epilimnion.

### Methane oxidation under oxic and anoxic conditions

The potential methane oxidation rates observed in Lake La Cruz (1.5–2.6 μM d^−1^) were of similar magnitude as reported estimates for other stratified lakes (e.g., Lidstrom and Somers, 1984; Blees et al., 2014). In shallow stratified lakes, highest methane turnover is commonly observed right at the oxycline (Panganiban et al., 1979; Sundh et al., 2005), where aerobic MOB have access to both oxygen and methane. In some cases, the MO potential at the oxycline was observed to be up to 60 times higher than above or below this interface (Carini et al., 2005; Schubert et al., 2010). This did not appear to be the case in Lake La Cruz where the methane turnover potential was similar under oxic and anoxic conditions.

Active MO in the apparent absence of O_2_ (15, 16, and 18 m) was supported by the δ^13^C-CH_4_ profiles exhibiting C isotopic fractionation of residual CH_4_ from and above 17 m (Figure 1C). We could verify sub-micro oxic conditions below 13.6 m and complete anoxia below 14.6 m with a trace oxygen optode with a detection limit of 20 nM (Figure 1A). Down to this depth, micro-aerobic MO could be supported (Blees et al., 2014). Yet below 14 m, the only known electron acceptor available in high enough *in situ* concentrations to account for measured MO potential was sulfate (35 μM). However, sulfate-coupled AOM seems to be very unlikely, as there was no visible consumption of ${\text{SO}}_{4}^{2-}$ or production of H_2_S within the zone of methane oxidation, and fluxes of both species were extremely low (Table 2). Furthermore, we did not observe any detectable characteristic shift to lighter δ^13^C-DIC values and lower pH (Supplementary Figure 1), which would be expected to result from AOM activity (Knittel and Boetius, 2009). Nor were targeted groups of known AOM-performing ANME or AAA found at any of the investigated depths, supporting previous evidence that these organisms are rare in lacustrine water columns (Eller et al., 2005; Oswald et al., 2016). Altogether, this suggests that classical AOM was not occurring in Lake La Cruz.

While true obligate anaerobic methanotrophs seemed to be absent, aerobic alpha- and gamma-proteobacterial MOB were detected at all investigated depths (12, 14, 15, 16, and 18 m; Figures 3, 4). The observed decrease in aerobic MOB cell abundances (2.5% at 12 m to 0.04% at 18 m) correlated with depth and oxygen availability suggesting a pivotal role of O_2_ in methane oxidation, as also observed in other lakes (Schubert et al., 2010; Zigah et al., 2015; Oswald et al., 2016). At least at 18 and 19 m 16S rRNA gene sequences belonging to the NC 10 phylum were detected (Figure 5). To some extent, members of the NC10 phylum may explain the occurrence of methane oxidation in the anoxic waters of Lake La Cruz in the absence of AOM-mediating archaea. However, as potential oxidation rates at 18 m were extremely low and given that neither ${\text{NO}}_{3}^{-}$ nor ${\text{NO}}_{2}^{-}$ are available at these depths under natural conditions throughout most of the year, we assume that these microorganisms do not contribute substantially to methane consumption in the lake.

### The role of oxygen and light in methane oxidation

Aerobic MOB in anoxic waters may be supported by a cryptic or transient supply of O_2_ below the oxycline. Indeed, methane oxidation was stimulated with the addition of oxygen (15 μM; Figure 6E). The resulting almost linear oxidation kinetics at 15 m, suggest an adequate supply of all substrates. In contrast, at the oxycline (14 m), the methane-oxidation time series displayed equivalent dynamics with and without added O_2_ (Figure 6D), suggesting that aerobic MO was limited by factors other than oxygen scarcity. Most likely, aerobic MOB at this depth were outcompeted by other, more abundant aerobic heterotrophs. The differences in total oxidized CH_4_ observed between 15 m (4.0 μM) and 16 m (2.2 μM) probably result from a six-fold higher MOB abundance at 15 m, leading to more efficient consumption with the same amount of supplied O_2_. Consequently, it appears that in the presence of O_2_, MO depends on the cell density of methanotrophs and the possible competition with other biotic (and possibly abiotic) oxidative processes.

While oxygen may be introduced into the hypolimnion of Lake La Cruz through sub-lacustrine springs after sporadic rainfalls, cryptic *in situ* oxygen production by low light photosynthesis may support aerobic MO throughout the year. Such photosynthesis-linked MO has been observed in other shallow lake systems with deep light penetration (Milucka et al., 2015; Oswald et al., 2015; Brand et al., 2016) and seems to be a plausible explanation for aerobic MO in the anoxic parts of Lake La Cruz. Laboratory and field evidence suggests that the lower PAR threshold for oxygenic photosynthesis in freshwater is 0.09 μE m^−2^ s^−1^ (Gibson, 1985) to 0.34 μE m^−2^ s^−1^ (Brand et al., 2016), respectively. Thus, light would have been sufficient to support photosynthesis well below the oxycline (0.1 μE m^−2^ s^−1^ at 16.5 m; Figure 1B), and could account for the apparent electron imbalance of ~5 mmol e^−^ m^−2^ d^−1^ (Table 2). Indeed, peaks in the chlorophyll a profiles indicate the presence of phototrophic organisms down to 18 m, and more importantly, light stimulated experimental methane consumption for all test depths (Figures 6D–F). Nevertheless, stimulation of MO was much less prominent in Lake La Cruz compared to previous studies (Milucka et al., 2015; Oswald et al., 2015) and appeared to slow down after ~12 h. For comparison, light promoted continuous linear oxidation (Milucka et al., 2015) and increased the MO potential by up to an order of magnitude (Oswald et al., 2015) in other shallow lakes. It is possible that primary production in Lake La Cruz was limited by the low nitrate (Figure 2A) and phosphate concentrations (average of 40 nM) throughout the whole water column. In any case, light-driven methane oxidation alone cannot fully explain all of the methane removal at and below the oxycline of Lake La Cruz.

### Alternative oxidants in methane oxidation

Although *in situ* concentrations of other potential electron acceptors [${\text{NO}}_{3}^{-}$, ${\text{NO}}_{2}^{-}$, Fe(III), and Mn(IV)] were too low to sustain the observed rates, their addition promoted MO to some degree at all depths (under dark conditions; Figure 6). Initial rates with supplemented nitrate or nitrite were only slightly higher (~2 μM d^−1^) than in the untreated dark control (1.5 μM d^−1^), yet both additions resulted in constant oxidation of methane throughout the incubation period with water from 14 m (i.e., the depth of the oxycline; Figure 6G). This steady turnover resulted in total oxidized CH_4_, which was about 3 (4.2 μM) and 2 (2.9 μM) times higher with supplemented nitrate and nitrite, respectively, compared to the dark control experiments (1.7 μM). Considering the stoichiometric ratio required for nitrate- (4:1) and nitrite- (8:3) coupled MO, the amount of nitrate (40 μM) and nitrite (20 μM) added to the incubation could explain observed rates. Stimulation of MO was much less pronounced at 15 and 16 m and bulk methane turnover was only slightly higher (max. 2.1 μM at 15 m with added nitrate) than in the control.

Simultaneous denitrification (${\text{NO}}_{3}^{-}$ and ${\text{NO}}_{2}^{-}$ supported) occurred in all incubations (data not shown). However, NO_x_ reduction rates and ^30^N_2_ production rates were considerably below (4–700 times lower; data not shown) what would be expected if it were directly linked to methane turnover. Part of this difference may be due to the parallel production of ^29^N_2_ with unlabeled substrates (mostly *in situ* produced NO_x_). It is also possible that NO_x_ reduction was partly balanced by nitrification, explaining the comparatively low net nitrate/nitrite reduction rates. Ongoing production by nitrifying bacteria, at least at the oxycline where O_2_ and ${\text{NH}}_{4}^{+}$ co-occur, could have been a constant source of both ${\text{NO}}_{3}^{-}$ and ${\text{NO}}_{2}^{-}$. This “hidden” nitrate/nitrite regeneration may have resulted in constant MO (linear production of ^13^CO_2_) without the corresponding apparent NO_x_ consumption. DOC concentrations to support organotrophic denitrification where quite high (40 μM; Supplementary Figure 1C; Richards et al., 1965), and, in contrast to the mid water depths where maximum CH_4_ turnover rates were observed, MO stimulation with NO_*x*_ was not observed at 15 and 16 m. These observations make it difficult to make a clear case for a link between MO and denitrification, at least in the deeper waters. However, we nevertheless cannot exclude the possibility that canonical denitrification and NO_x_-dependent MO co-occur.

We did not specifically test for nitrite-reducing *M. oxyfera* (Ettwig et al., 2010) or nitrate-reducing ANME-2d (Raghoebarsing et al., 2006; Haroon et al., 2013) via CARD-FISH. However, as 16S rRNA gene sequences related to the NC 10 phylum were only retrieved below the zone of high methane oxidation potential, where NO_x_ is lacking (18 and 19 m; Figure 5) and other representatives of ANME (−1 and −2) and AAA were absent, it is unlikely that either group contributes to methane turnover in Lake La Cruz. We found, however, microbiological/molecular (i.e., CARD-FISH, sequencing) evidence for the presence of aerobic gamma-MOB at anoxic depths (Figures 3, 4). It has been shown recently that some of these bacteria can couple methane oxidation to nitrate/nitrite reduction under oxygen limitation, although trace amounts of oxygen are probably still required for the initial oxidation of methane to methanol (Kits et al., 2015a,b). This metabolic switch would provide gamma-MOB with a competitive advantage in an environment with fluctuating O_2_ conditions (i.e., transient sub-micromolar O_2_ concentrations) and could explain why zones of aerobic MO and nitrate/nitrite-dependent methane oxidation appear to overlap in the environment (Deutzmann et al., 2014). A facultative aerobic MO mechanism could also explain why MO was simultaneously stimulated by O_2_, NO_x_, and light herein.

Presently, we can only speculate about any direct coupling of MO and denitrification. Indirect stimulation by nitrate and nitrite must also be considered. Most MOB either fix nitrogen (Davis et al., 1964) or derive it from an inorganic source to build up their biomass (Rudd et al., 1976; Bodelier and Laanbroek, 2004). The addition of an inorganic nitrogen source may simply have remedied N limitation, thus enhancing N uptake and growth of aerobic MOB. In this case the electron acceptor involved in the oxidation of methane would remain unknown.

Theoretically, Mn(IV) and Fe(III) could serve as the electron acceptor for MO although the organisms performing this reaction have yet to be identified (Crowe et al., 2011; Sivan et al., 2011; Norði et al., 2013). Since aerobic gamma-MOB are, with regards to their metabolic requirements, more versatile than previously believed (Kits et al., 2015a,b), it is conceivable that both Mn(IV) and Fe(III) could serve as viable electron acceptors in the respiratory chain, in addition to O_2_ and nitrate or nitrite (Kits et al., 2015a,b).

While the addition of birnessite and ferrihydrite did appear to increase MO rates and methane turnover at some depths, we were not able to establish clear links between observed MO and concurrent metal reduction. In experiments with birnessite, dissolved (reduced) Mn concentrations did not increase over time (data not shown). In experiments with ferrihydrite, we can only speculate about whether iron reduction really occurred. In the 14 and 15 m incubations, the Fe(III) concentrations did not show any decreasing trend (data not shown). At 16 m, Fe(III) decreased by 5% (or 2.4 μM), yet the observed concentration change was insufficient (by a factor of 5) to explain observed methane consumption. It is important to note, however, that produced Fe(II) can be re-oxidized by a variety of abiotic (O_2_ and MnO_2_) and biotic oxidative processes (i.e., Fe-oxidizing bacteria; Melton et al., 2014). Whereas, both O_2_ and MnO_2_ were scarce at these depths, the activity of phototrophic and nitrate-dependent Fe-oxidizers could have continuously recycled reduced Fe in deeper waters of the lake (Sobolev and Roden, 2002; Emerson, 2009; Bruun et al., 2010). Both phototrophic (Walter et al., 2014) and nitrate-dependent Fe-oxidizers (Walter, 2011) have been found in Lake La Cruz, yet phototrophic recycling of Fe was not possible in our dark experiments. Likewise, it is questionable whether the ${\text{NO}}_{3}^{-}$ concentrations were sufficient to maintain continuous iron oxidation. Similarly, to NO_x_, the addition of Mn(IV) or Fe(III) may also have indirectly stimulated MO, especially at 14 m. It is possible that MnO_2_ in our incubations oxidized *in situ* ${\text{NH}}_{4}^{+}$ (e.g., Luther et al., 1997), supplying ${\text{NO}}_{3}^{-}$ as an oxidant for MO or as an inorganic N source for MOB. Iron is an important trace metal for methanotrophs (Semrau et al., 2010; Glass and Orphan, 2012) and iron addition thus could also enhance their activity (without being exploited as electron acceptor).

In conclusion, our findings suggest that methane emissions from Lake La Cruz are effectively mitigated by methane oxidation both in oxic and anoxic waters. Under both oxic and micro-oxic conditions, aerobic MOB utilize oxygen as the oxidant. Under anoxic conditions, aerobic methane turnover is most likely supported through the coupling with oxygenic photosynthesis. In addition, we found evidence that electron acceptors besides oxygen, especially ${\text{NO}}_{3}^{-}$ and ${\text{NO}}_{2}^{-}$, stimulate methane consumption at the oxycline. Potential direct links between MO and the reduction of other alternative electron acceptors and at other depths remain inconclusive and require further investigation. Aerobic MOB alone appear to be responsible for methane removal in the La Cruz water column, though their actual activity remains to be quantified (i.e., with nanoSIMS). Although, we cannot completely rule out the presence of yet unknown anaerobic methanotrophs, our data provide putative evidence for non-archaeal methane oxidation under anoxic conditions in an aquatic environment. Future research in Lake La Cruz should focus on further characterizing the methanotrophic community and activity, with particular focus on aerobic MOB that are also known to oxidize methane with nitrate and nitrite (e.g., *Methylomonas denitrificans* and *Methylomicrobium album*).

## Author contributions

KO, CJ, JT, JB, AB, MM, XS, EV, ML, and JZ were involved in sampling preparation, field sampling and field experiments. MM, XS, and EV coordinated the logistics and sampling equipment on site. CJ, JT, JB, XS, and KO conducted laboratory and data analyses. AB, MM, XS, and EV analyzed and provided oxygen and chlorophyll a data. CS, ML, JZ and KO designed the study and experimental setup. KO, CJ, AB, ML, JZ, and CS contributed to data evaluation and interpretation. KO and CJ wrote the manuscript. JT, JB, AB, MM, XS, EV, ML, JZ, and CS helped improve the manuscript with their valuable comments and critical feedback.

## Funding

This study was part of a larger research effort “Methane oxidation pathways at oxic-anoxic boundaries in lakes” and funded by SNF grants: 135299, 153091, and 128707. Further funding for material and equipment was provided by Eawag, University of Basel, University of Valencia.

### Conflict of interest statement

The authors declare that the research was conducted in the absence of any commercial or financial relationships that could be construed as a potential conflict of interest.
