# Supplementary material for: Methanotrophy under Versatile Conditions in the Water Column of the Ferruginous Meromictic Lake La Cruz (Spain)
Source: Front Microbiol. 2016 Nov 11;7:1762. doi: 10.3389/fmicb.2016.01762 (PMC5104750; doi:10.3389/fmicb.2016.01762)
Supplement: Supplementary file 1 [file DataSheet1.PDF]

Supplementary Material

Methanotrophy under versatile conditions in the water column of the ferruginous meromictic Lake La Cruz (Spain)

Kirsten Oswald*, Corinne Jegge, Jana Tischer, Jasmine Berg, Andreas Brand, María Rosa Miracle, Xavier Soria, Eduardo Vicente, Moritz F. Lehmann, Jakob Zopfi & Carsten J. Schubert*

*Correspondence: kirsten.oswald@eawag.ch & carsten.schubert@eawag.ch

Supplementary Figures

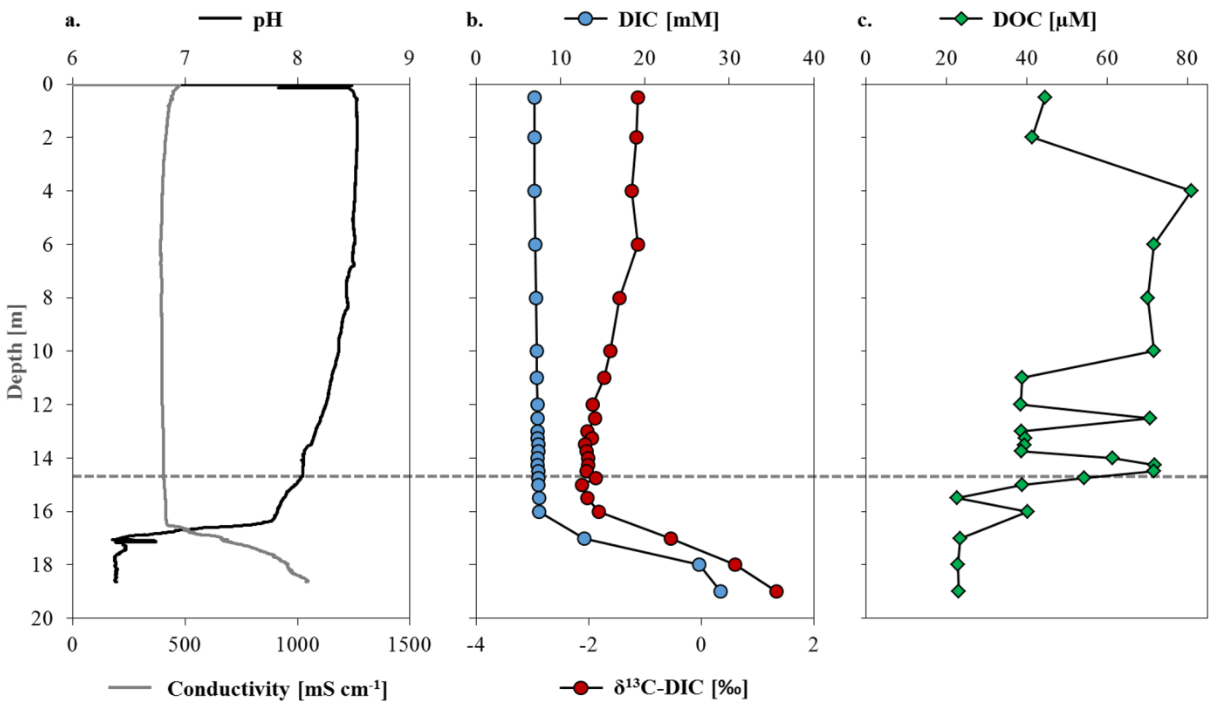

Supplementary Figure 1. Additional physical and chemical parameters in the Lake La Cruz water column. (a) Profiles of pH and conductivity; (b) dissolved inorganic carbon concentrations and the corresponding δ^{13}C-DIC; and (c) dissolved organic carbon concentrations. The dashed line indicates the depth of the oxycline.

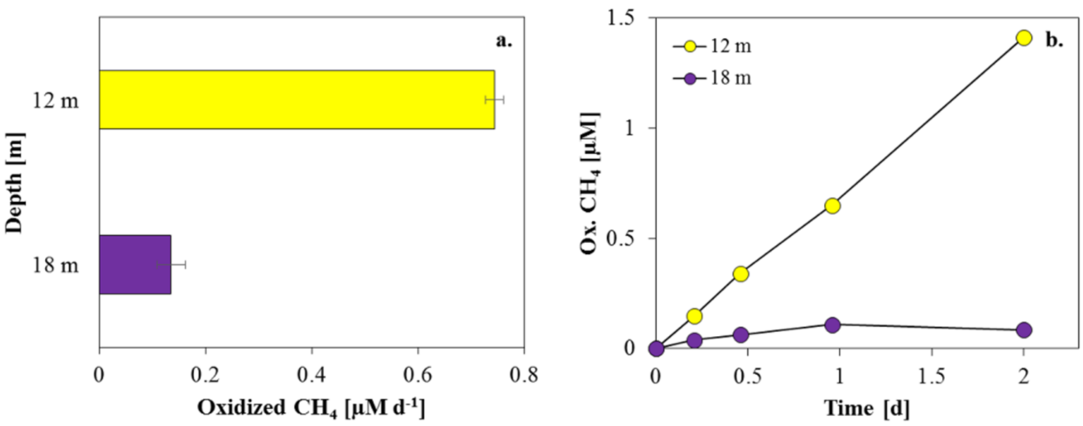

Supplementary Figure 2. Methane oxidation potential measured at 12 and 18 m. (a) Initial methane oxidation rates calculated over the initial linear segment (~12h) of the corresponding time series (b).

Supplementary Tables

Supplementary Table 1. Tested CARD-FISH probes along with their specificity, amount of formamide in the hybridization buffer, probe sequence and the appropriate reference.

Probe	Specificity	Formamide	Probe sequence (5´-3´)	Reference
AAA-FW-641	AOM-associated archaea	60%	GGT CCC AAG CCT ACC AGT	Schubert et al. (2011)
AAA-FW-834			TGC GGT CGC ACC GCA CCT	
ANME-1-350	ANME-1	40%	AGT TTT CGC GCC TGATGC	Boetius et al. (2000)
ANME-2-538	ANME-2	40%	GGC TAC CAC TCG GGC CGC	Treude et al. (2005)
EUB338 I-III	Most bacteria	35%	GCT GCC TCC CGT AGG AGT<br>GCA GCC ACC CGT AGG TGT<br>GCT GCC ACC CGT AGG TGT	Daims et al. (1999)
Ma450	Alpha-MOB	20%	ATC CAG GTA CCG TCA TTA TC	Eller and Frenzel (2001)
Mgamma84	Gamma-MOB	20%	CCA CTC GTC AGC GCC CGA	Eller and Frenzel (2001)
Mgamma705			CTG GTG TTC CTT CAG ATC	
NON338	Negative control	35%	ACT CCT ACG GGA GGC AGC	Wallner et al. (1993)

Supplementary References

Boetius, A., K. Ravenschlag, C. J. Schubert, D. Rickert, F. Widdel, A. Gieseke et al. (2000). A marine microbial consortium apparently mediating anaerobic oxidation of methane. Nature 407: 623-626. doi: 10.1038/35036572.

Daims, H., A. Brühl, R. Amann, K.-H. Schleifer, and M. Wagner. (1999). The domain-specific probe EUB338 is insufficient for the detection of all bacteria: development and evaluation of a more comprehensive probe set. Systematic and Applied Microbiology 22: 434-444. doi: 10.1016/S0723-2020(99)80053-8.

Eller, G., and P. Frenzel. (2001). Changes in activity and community structure of methane-oxidizing bacteria over the growth period of rice. Applied and Environmental Microbiology 67: 2395-2403. doi: 10.1128/AEM.67.6.2395-2403.2001.

Schubert, C. J., F. Vazquez, T. Lösekann‐Behrens, K. Knittel, M. Tonolla, and A. Boetius. (2011). Evidence for anaerobic oxidation of methane in sediments of a freshwater system (Lago di Cadagno). FEMS Microbiology Ecology 76: 26-38. doi: 10.1111/j.1574-6941.2010.01036.x.

Treude, T., J. Niggemann, J. Kallmeyer, P. Wintersteller, C. J. Schubert, A. Boetius et al. (2005). Anaerobic oxidation of methane and sulfate reduction along the Chilean continental margin. Geochimica et Cosmochimica Acta 69: 2767-2779. doi: 10.1016/j.gca.2005.01.002.

Wallner, G., R. Amann, and W. Beisker. (1993). Optimizing fluorescent in situ hybridization with rRNA‐targeted oligonucleotide probes for flow cytometric identification of microorganisms. Cytometry 14: 136-143. doi: 10.1002/cyto.990140205.
